# A large deviation principle for networks of rate neurons with correlated synaptic weights

**DOI:** 10.1186/1471-2202-14-S1-P252

**Published:** 2013-07-08

**Authors:** Olivier Faugeras, James MacLaurin

**Affiliations:** 1 NeuroMathComp Laboratory, INRIA Sophia-Antipolis, France

## 

One of the major goals of mathematical neuroscience is to rigorously justify macroscopic continuum neural-field equations by deriving them from microscopic equations governing the interactions of individual neurons. Since the macroscopic variables are continuous, their value at a particular point is usually interpreted as a mean taken over the neurons in a small neighborhood of the point. In order that the mean is an accurate approximation, it is normally assumed / proved that the neurons are approximately uncorrelated, so that the law of large numbers implies that their average behavior is close to the mean. We develop a model of neural networks with inhomogeneous weights between the neurons, and analyze its behavior as the number of neurons asymptotes to infinity. It will be seen that the inhomogeneity of the weights ensures that the neurons in the limit system are not uncorrelated. Our results thus suggest that the mean-field approximation is insufficient.

We study the asymptotic behavior of a network of *N *firing rate neurons as the number *N *grows to infinity. The neurons are modeled as lying equally-spaced on a ring. The membrane potential of each neuron evolves according to a discrete time version of the Hopfield or Wilson-Cowan equations [1]. The synaptic weights *J(i,j) *of presynaptic neuron *j *and postsynaptic neuron *i *are modeled as Gaussian Random variables, with identical means that scale as one over *N*. The covariance between *J(i,j) *and *J(k,l) *also scales as one over *N *times *C(i-k,j-l) *for some fixed function *C*. In other words, the covariance is considered to be a function of the 'ring distance' between the presynaptic and postsynaptic.

Our main result is that the behavior of the infinite size ensemble of neurons can be described by a simple nonlinear transformation of a spatially stationary (along the ring) Gaussian random process. The nonlinearity is a combination of the firing rate function and the leak. This Gaussian process is described by its mean, the same time-varying function for each neuron, and its covariance operator. The covariance operator describes the correlation between any *k*-tuple of neurons. It is also stationary in the sense that if we translate each neuron of the *k*-tuple by the same amount along the ring, the correlation does not change. We have been able to obtain explicitly the equations that describe the mean and covariance of the limit Gaussian process. They form a set of strongly coupled recursive (in time) equations.

Our analysis goes beyond the identification of the asymptotic limit of the network. We also prove that in effect the probability law that describes the solutions to the network equations converges exponentially fast toward the previous limit (in a precise mathematical sense) and we have been able to compute the specific rate of convergence thanks to the use of the theory of Large Deviations [2]. This rate of convergence is given explicitly by a function (called the good rate function) defined over the set of all possible asymptotic limit probability laws. We prove that it has a unique minimum at the asymptotic limit.

Most modeling of neural networks assumes / proves some sort of thermodynamic limit, whereby if one isolates a particular population of neurons in a localized area of space, they are found to fire increasingly asynchronously as the number in the population asymptotes to infinity, e.g. [3]. However our limit does not possess this property: the nontrivial covariances between the weights ensures that there are large system-wide correlations between the neurons in the asymptotic limit. A very important implication of our result is that the mean-field behavior is insufficient to characterize the behavior of a population. Our work challenges the assumption held by some that one cannot have a concise macroscopic description of a neural network without an assumption of asynchronicity at the local population level. It is a generalization of the work of Moynot and Samuelidies [4].
